# Characteristic Temporary Loss of Taste and Olfactory Senses in SARS-CoV-2-positive-Individuals with Mild Symptoms

**DOI:** 10.20411/pai.v5i1.374

**Published:** 2020-05-16

**Authors:** Ricarda M. Schmithausen, Manuel Döhla, Heidrun Schöβler, Christin Diegmann, Bianca Schulte, Enrico Richter, Anna-Maria Eis-Hübinger, Hendrik Streeck

**Affiliations:** 1 Institute for Hygiene and Public Health; One Health Department; Medical Faculty; University of Bonn; Venusberg Campus 1; 53127 Bonn, Germany; 2 Local Health Authority; Heinsberg; Valkenburger Str. 45; 52525 Heinsberg, Germany; 3 Institute of Virology; Medical Faculty; University of Bonn; Venusberg Campus 1; 53127 Bonn, Germany; # contributed equally

**Keywords:** Coronavirus, SARS-CoV-2, COVID-19, Symptoms

SARS-CoV-2 infection has been characterized as an upper and lower respiratory tract infection with symptoms ranging from sore throat, cough, headache, and fatigue to a severe respiratory syndrome that requires intensive care [1–7]. Although a lower death rate has been recorded for SARS-CoV-2 in comparison to other recent coronavirus outbreaks, such as MERS or SARS, a compromised respiratory status on admission has been associated with fatal outcomes. Among the >1200 SARSCoV-2-infected individuals in Germany to date, many of the diagnosed persons show mild symptoms to no clinical signs of infection. Here we describe the onset and characteristics of symptoms in a cluster outbreak after a carnival celebration in a small town in the state of North Rhine-Westphalia currently contributing 43% of the total number of infected individuals in Germany to date [8]. Among the nearly 500 individuals in domestic quarantine as ordered by the Local Health Authority at that time, we interviewed 41 randomly selected individuals with qPCR confirmed SARS-CoV-2 infection. Inclusion criteria for the survey participation were: residents, positive for SARS-CoV-2 in pharyngeal swabs, ≥18 years of age, and confirmed informed consent. Median age was 40 years (IQR: 31-53, range: 18-82) and 51%were female. All persons examined had attended a carnival festivity also attended by the SARS-CoV-2positive index patient. All persons who attended this carnival festivity were tested for SARS-CoV-2 by the local health authorities 10 days later. Among survey participants, onset of symptoms began 12 days (IQR: 11-13) after probable infection and a median of 6 distinct symptoms (IQR: 4-8) were described.

The most frequent symptom was a dry and persistent, non-productive cough, which was present in 30 individuals (73%). Interestingly, 12 (IQR: 12-14.5) days after probable infection, individuals described a disturbing loss of taste and/or olfactory sense (28 patients, 68%). Onset of dysosmia and dysgeusia was described as gradual, persisting for 3-5 days, and followed by a slow recovery. There was no association with any other symptoms, such as sore throat, runny nose, or headache. Dysosmia is also described for different viral infections (sinusitis, common cold), but also as a symptom of other diseases (stroke, drug abuse, tumors, craniocerebral trauma, toxin poisoning) [9]. Accordingly to our data, other recent studies also show that dysosmia could be an independent predictive symptom of COVID-19 [10–13]. Another common symptom was general fatigue (28 patients, 68%), followed by runny nose (21 patients, 51%), and headache and muscle/limb pain (18 patients each, 44%). Seventeen patients had fever (42%), 17 patients had a sore throat (42%), and one third described persistent diarrhea (13 patients, 32%), which was more frequent than in other reports.

Although previous reports have mainly focused on moderate to severe symptoms, we describe here distinct and characteristic symptoms in individuals with only mild symptoms. Thus, our data suggest that individuals with a combination of a dry cough and loss of smell and/or taste should trigger diagnostic evaluation for SARS-CoV-2.
